# Nystatin-mediated bismuth oxide nano-drug synthesis using gamma rays for increasing the antimicrobial and antibiofilm activities against some pathogenic bacteria and *Candida* species

**DOI:** 10.1039/c9ra10765g

**Published:** 2020-03-05

**Authors:** Ahmed I. El-Batal, Hanady G. Nada, Reham R. El-Behery, Mohamed Gobara, Gharieb S. El-Sayyad

**Affiliations:** Drug Radiation Research Department, National Centre for Radiation Research and Technology (NCRRT), Egyptian Atomic Energy Authority (EAEA) P. O. Box 29, Nasr City Cairo Egypt Gharieb.Elsayyad@eaea.org.eg hanady.nada@eaea.org.eg; Chemical Engineering Department, Military Technical College (MTC), Egyptian Armed Forces Cairo Egypt

## Abstract

The novelty of the present research is the synthesis of bismuth oxide nanoparticles (Bi_2_O_3_ NPs) loaded with the antifungal nystatin drug *via* gamma rays for increased synergistic antimicrobial potential against some pathogenic bacteria and *Candida* species. The full characterization of the synthesized Bi_2_O_3_ NPs-Nystatin was achieved by XRD, FT-IR, HR-TEM, and SEM/EDX mapping techniques in order to analyze the crystallinity, chemical functional groups, average particle size, morphology, and elemental structure, respectively. The antimicrobial activities of Bi_2_O_3_ NPs-Nystatin were examined against pathogenic bacteria and *Candida* species, including the zone of inhibition (ZOI), minimum inhibitory concentration (MIC), and antibiofilm activity. Additionally, the SEM/EDX method was performed to investigate the mode of action on the treated *Candida* cells. Our results revealed that Bi_2_O_3_ NPs-Nystatin possessed a well-crystallized semi-spherical shape with an average particle size of 27.97 nm. EDX elemental study of the synthesized Bi_2_O_3_ NPs-Nystatin indicated a high level of purity. Interestingly, the synthesized Bi_2_O_3_ NPs-Nystatin displayed encouraging antibacterial behavior against almost all the tested bacteria and a synergistic antifungal potential toward the investigated *Candida* species. Additionally, Bi_2_O_3_ NPs-Nystatin was found to be a promising antibiofilm agent, resulting in inhibition percentages of 94.15% and 84.85% against *C. albicans* (1) and *E. coli*, respectively. The present research provides a revolutionary nano-drug-based solution to address the increasing global resistance of pathogenic microbes at low concentrations, thus offering a new infectious disease treatment technique that is cost effective, eco-friendly, and works in an acceptable time frame.

## Abstract 2

The four common mechanisms of the antimicrobial activity of Bi_2_O_3_ NPs-Nystatin.

## Graphical abstract

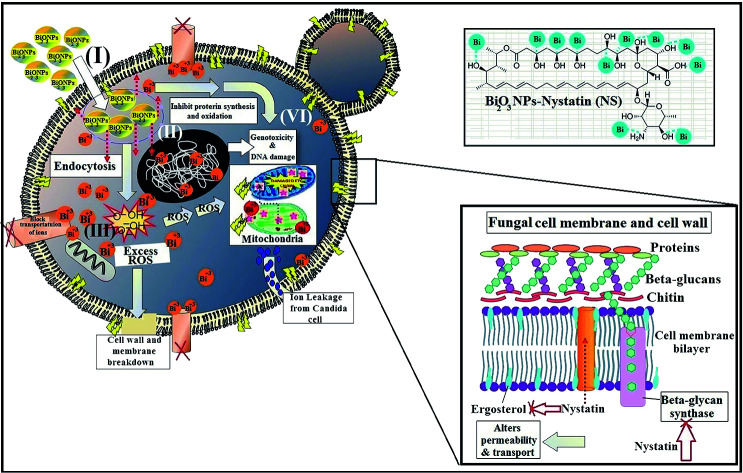

## Introduction

Like many microbes, some fungi live naturally as commensals inside human and animal bodies. However, fungal diseases happen when a pathogenic fungus invades a patient's body and affects their immune system.^1^ The most common fungi that possess the capability to create life-threatening infections include *Candida albicans* and *Aspergillus fumigates*.^2^*Candida* species are classified as the fourth most common reason for clinical and systemic diseases in the USA, with aggressive death rates of up to 55%.^3^

*C. albicans* is categorized as an opportunistic pathogen that can also be observed as a part of the normal microflora in the digestive systems of animals and humans.^4^ However, a slight change in the host protection system can support the conversion of *C. albicans* into a pathogen that is capable of producing diseases.^5^ They can create two major kinds of diseases in humans: surface diseases (oral or vaginal candidiasis) and life-endangering systemic diseases.^6^ There are some determinants willing to enhancing the virulence of *Candida* species such as the expanded usage of complete parenteral diet, intravenous catheters, broad-spectrum antibiotics, and cytotoxic chemotherapy.^7^

Tremendous progress in fungal diagnostics and antifungal medication has been made in the past 25 years; however, this has not been reflected in significant changes in antifungal production.^8^ The emergence of *C. albicans* has highlighted some real challenges because several species present different levels of resistance (acquired or natural), creating difficulties for the generally applied antifungal drugs to work effectively.^9^ The elevated number of drug-resistant fungal pathogens and the toxicity of the present antifungal composites have focused significant attention to the antimicrobial potential of biogenic nano-based composites.^10^

It must be noted that a small number of antifungal agents have been prepared for use in yeast treatment, most of which are considered as fungistatic. One of the key challenges associated with the treatment of bacterial and fungal infections with conventional drugs is that a tremendous resistance to antimicrobial drugs can occur, which is thus driving the research for alternative therapies.^11^ There are some articles in the literature^12,13^ regarding some examples of candidosis that were found to be clinically resistant to nystatin therapy, which makes the treatment of some *Candida* sp. by nystatin very difficult and requires nystatin to be incorporated into a nano-carrier.

Nanotechnology is an actively emerging discipline with great application potential in many fields, including pharmaceutics, chemistry, plant pathology, and biomedicine, and refers to materials with very small dimensions (at the atomic or molecular scales). In particular, in biomedical applications, a bio-nanotechnique is a proper method for eliminating or minimizing the attack of different pathogenic bacteria and fungi by administering a nano-drug.^14^ Many synthetic methods, like thermal reduction^15^ and biological synthesis,^14^ have been used to make metal oxide NPs.^16^

There is an increasing need for the green synthesis of metal oxide NPs for their application in pharmaceutical and biomedical fields due to their superior chemical, physical, and catalytic properties. Due to the encouraging characteristics of the prepared metal oxide NPs, they have found possible applications in biomedicine as anticancer and antimicrobial agents.^17^

However, the interactions between metal oxide NPs and biological systems depend on the cell type and uptake routes or the direction of different organelles. Despite the evolution of research in nanotechnology, there remain considerable challenges to overcome, including safety, scale-up production, decreasing costs, and understanding the biological activity.^18^ Nowadays, the safety concerns of using metal oxide NPs are considered one of the main future challenges for biomedical applications.^18^ Here, we tried to decrease the toxicity of the prepared metal oxide NPs by incorporation with a resistant-nystatin drug to increase the synergistic effect and decrease the necessary applied-dose in order to reduce the nanotoxicity, which means reducing the negative influence of the metal oxide NPs on the biological organisms.^18,19^

Bi_2_O_3_ NPs have a large surface area with various electrochemical balances and they have delivered important attention due to their potential applications for zinc sensing.^20^ Owing to their unique properties (non-toxic behavior, biocompatibility, and high chemical stability), they have been used as antibacterial and antifungal agents.^21^ There are some research reports on the antibacterial and antifungal activities of Bi_2_O_3_ NPs, including from Hernandez *et al.*,^22^ who investigated the fungicidal activity of Bi_2_O_3_ NPs against *C. albicans* as well as their antibiofilm capabilities, and showed that the Bi_2_O_3_ NPs displayed antimicrobial activity against *C. albicans* growth, reducing the colony size by 85%, and effected a complete inhibition of biofilm formation. Also, El-Batal *et al.*^23^ synthesized green Bi_2_O_3_ NPs from melanin pigment using gamma rays and examined their antimicrobial activity against some standard pathogenic bacteria and *Candida* sp. Their results indicated that the Bi_2_O_3_ NPs were active against *Escherichia coli* (13.0 mm ZOI), *Staphylococcus epidermidis* (23.0 mm ZOI), and *C. albicans* (20.0 mm ZOI).

Herein, we synthesized the nano-drug Bi_2_O_3_ NPs-Nystatin by gamma rays, which serves an eco-friendly and cost-efficient method. Full characterization techniques were performed to demonstrate the various properties of the synthesized Bi_2_O_3_ NPs-Nystatin. The synthesized Bi_2_O_3_ NPs-Nystatin possessed a small size, high crystallinity, complete elemental distribution, simplicity, and acceptable purity, which in turn led to elevated antimicrobial and antibiofilm activities. The significance of our results and findings is in the possible application of a new nano-drug synthesized by a green method at low concentration (to avoid the nanotoxicity and to reduce the used nystatin dose), which increases the synergistic potential of the nystatin drug against pathogenic microbes.

## Materials and methods

### Chemicals and reagents used

The media components were purchased from Hi-Media and Difco. The chemicals, such as bismuth nitrate, nystatin (NS), polyvinylpyrrolidone (PVP), dimethyl sulfoxide (DMSO), isopropyl alcohol, and other reagents used in the following tests (biological procedures) were utilized at the analytical grade as purchased from Sigma-Aldrich.

### Radiation source

The gamma-irradiation process was conducted at the NCRRT, Cairo, Egypt. The source of radiation was the 60Co-Gamma chamber 4000-A-India. The applied dose rate was fixed at 2.02 kGy h^−1^. Gamma rays produce a free radical and solvated electrons after water radiolysis.

### Synthesis and optimization of Bi_2_O_3_ NPs by the nystatin drug and gamma rays

Bi_2_O_3_ NPs were synthesized and incorporated the nystatin drug *via* applying gamma rays (as a reducing agent) in the presence of a capping polymer, like polyvinylpyrrolidone (PVP). Briefly, in a 10 ml test tube, a solution of 25.0 mg of bismuth nitrate and 1.0% PVP was mixed with 0.5 ml isopropyl alcohol and completed with distilled water to make up to a net volume of 9.0 ml aqueous solution (A). After that, 5.0 mg nystatin drug was dissolved in 1.0 ml dimethyl sulfoxide (DMSO) to form an aqueous solution (B). Finally, solutions A and B were mixed at room temperature (24.0 ± 2.0 °C) to a final ratio of 1 : 5 (nystatin : bismuth nitrate; v/v).

The resulting mixtures were irradiated by different gamma-ray doses (1.0, 3.0, 5.0, 10.0, 15.0, 20.0, 25.0, and 30.0 kGy). The optical characterization of the synthesized Bi_2_O_3_ NPs-Nystatin was performed by UV-Vis spectroscopy (JASCO V-560-UV-Vis spectrophotometer) with respect to a negative control (the gamma-irradiated sample without bismuth nitrate).

A gamma-ray dose with high optical density (O.D.) was selected for further investigation. Additionally, the stability of the nystatin drug was examined after exposure to different gamma-rays doses (as mentioned-before).

A preliminary investigation was carried out to define the impact of the bismuth nitrate and nystatin drug concentrations regarding the Bi_2_O_3_ NPs production (after the exposure to the most-effective gamma-ray dose). The expected factorial investigation included two factors, *i.e.*, bismuth nitrate and nystatin concentrations, over 12 levels (see Table 1). It must be noted that the main idea for the chosen factors used in the present study is that they had multiple significant influences on the Bi_2_O_3_ NPs production.

**Table 1:** Experimental factorial design for the optimization of Bi_2_O_3_ NP production using nystatin drug (NS) and bismuth nitrate after exposure to gamma rays at 20.0 kGy, their wavelength (nm), and the corresponding optical density

Runs	Nystatin (NS) (mg/10 ml)	Bismuth nitrate (mg/10 ml)	Ratio (mg; w/w)	Absorption of Bi_2_O_3_ NPs (O.D.)	Wavelength of Bi_2_O_3_ NPs (*λ* nm)
1	5.0	0.0	1 : 0	Control^a^	Control^a^
2	5.0	5.0	1 : 1	Nil^b^	Nil^b^
3	5.0	12.5	1 : 2.5	Nil^b^	Nil^b^
4	5.0	25.0	1 : 5	2.253	570.0
5	5.0	37.5	1 : 7.5	1.887	310.0
6	5.0	50.0	1 : 10	1.462	315.0
7	0.0	25.0	0 : 5	Control^a^	Control^a^
8	2.5	25.0	0.5 : 5	2.179	540.0
9	5.0	25.0	1 : 5	2.118	570.0
10	7.5	25.0	1.5 : 5	1.666	620.0
11	10.0	25.0	2 : 5	Nil^b^	Nil^b^
12	15.0	25.0	3 : 5	Nil^b^	Nil^b^

a Control was used to autozero the results from the set of other experiments that did not contain one of the precursors used for Bi_2_O_3_ NPs-Nystatin synthesis.

b Nil means there was no color change which confirms non-formation of Bi_2_O_3_ NPs-Nystatin.

Bismuth nitrate solution (at different concentrations; see Table 1) was mixed with different concentrations of nystatin drug solution in addition to 0.2% isopropyl alcohol. The prepared solutions were stirred at room temperature (24.0 ± 2.0 °C) and finally exposed to varying gamma-ray doses (as determined from the last investigation).

### Characterization of the synthesized Bi_2_O_3_ NPs-Nystatin

The crystallite sizes and the crystallinity of the synthesized Bi_2_O_3_ NPs-Nystatin were determined by XRD (XRD-6000, Shimadzu apparatus, SSI, Japan). The strength of the diffracted X-rays was recognized as per the diffraction angle 2*θ*.

The common size and particle-size distribution of the Bi_2_O_3_ NPs-Nystatin were defined by dynamic light scattering (DLS-PSS-NICOMP 380-USA). Additionally, the average nano-structure and the particle size of the synthesized Bi_2_O_3_ NPs-Nystatin were determined by high-resolution transmission electron microscopy (HRTEM, JEM2100, Jeol, Japan).

The surface and morphological features were examined by scanning electron microscopy (SEM, ZEISS, EVO-MA10, Germany). Also, EDX spectrum examination (BRUKER, Nano GmbH, D-12489, 410-M, Germany) was used to estimate the elemental composition, purity, and the relationship of each metal. SEM/EDX mapping method was applied for obtaining further information regarding the structure/simplicity, relationships, and the position of the metals in the synthesized Bi_2_O_3_ NPs-Nystatin.

Finally, FT-IR spectroscopy was performed to provide important data about the chemical functional groups present on the nystatin drug. The analyses were carried out using a JASCO FT-IR 3600 infra-red spectrometer and by using the KBr pellet method. It was determined at a wavenumber scale from 4000 to 400 cm^−1^.

### Antimicrobial activity of the synthesized Bi_2_O_3_ NPs-Nystatin

The antimicrobial activities of the synthesized Bi_2_O_3_ NPs-Nystatin, polyvinylpyrrolidone (PVP), dimethyl sulfoxide (DMSO), nystatin drug, and bismuth ions were tested against some selected *Candida* species and pathogenic bacteria using the agar disc distribution method.^24^

The examined microbes were taken from the culture collections at the Drug Microbiology Laboratory, Drug Radiation Research Department, NCRRT, Cairo, Egypt. The tested unicellular fungi were *Candida albicans* (1), *Candida tropicalis* (1), *Candida tropicalis* (22), *Candida albicans* (25), and *Candida albicans* (33), while the pathogenic bacteria included Gram-positive (*Bacillus cereus* and *Staphylococcus aureus*; MRSA) and Gram-negative bacteria (*Escherichia coli* and *Pseudomonas aeruginosa*).

The tested bacterial inoculums were fixed with 0.5 McFarland (2–4) × 10^7^ CFU ml^−1^, and all the examined *C. albicans* and *C. tropicalis* were fixed with 0.5 McFarland (3–5) ×10^8^ CFU ml^−1^ after conducting UV-Vis spectrophotometry at 600 nm.^25^

Nystatin antifungal disc (NS 100; 100 μg ml^−1^) and amoxicillin/clavulanic acid (AMC; 20/10 μg ml^−1^) were examined as standard antibiotics. The growth restraint of the examined microbes was confirmed by the zone of inhibition (ZOI) after 24 h incubation.^14^

The minimum inhibitory concentration (MIC) was assessed referring to the lowest concentration of Bi_2_O_3_ NPs-Nystatin that inhibited 99.0% of the bacterial and yeast growth. For this, the serial dilution method of a Luria–Bertani (LB) broth medium containing the tested microbes was applied using the ELISA plate method.^26^ The inoculums were fixed as mentioned-before in the first antimicrobial screening.^25^ Broth medium, DMSO, and PVP were used as a negative control, while the standard antibiotics (AMC and NS) were used as a positive control. Finally, serial dilutions of Bi_2_O_3_ NPs-Nystatin (starting with a concentration of 250 μg ml^−1^) were used. All the plates were incubated for 24 h at 36.0 ± 1.0 °C and examined at 600 nm.^14^

### Antibiofilm activity of the synthesized Bi_2_O_3_ NPs-Nystatin

A qualitative study of the biofilm inhibition was performed as described by Christensen *et al.*^27^ Biofilm formation across the tube walls in the absence and presence of the synthesized Bi_2_O_3_ NPs-Nystatin was investigated.

The antibiofilm potential of the synthesized Bi_2_O_3_ NPs-Nystatin (at a ratio of 1 : 5 w/w) was examined against the most sensitive bacteria and *Candida* sp. with respect to the non-treated strain (control).

Briefly, 5.0 ml of the nutrient broth medium was poured in to the tubes and the tested pathogenic bacteria and yeast cells were inoculated with 0.5 McFarland adjusted from 1–3 × 10^7^ CFU ml^−1^. Subsequently, they were incubated for 24 h at 35.0 ± 2.0 °C, and after that, the supernatant was discharged and all the tubes were mixed with phosphate buffer saline (PBS; pH 7.0) and finally dried.^28^

The biofilms around and at the bottom of the tube walls were rinsed with 3.0 ml sodium acetate (3.5%) for 15 min. and subsequently washed with de-ionized water. After that, 10.0 ml crystal violet (CV; 0.1%) was added to the biofilms that had developed inside the tubes (about 30 min.) for the staining and finally the tubes were cleaned with de-ionized water.^29^

It is worth mentioning that, for the semi-quantitative antibiofilm investigation (inhibition%), 3.0 ml of the absolute ethanol was connected to the tubes to separate the colored biofilms,^30^ and after that, the O.D. of CV was measured using a UV-Vis spectrophotometer at a fixed wavelength (570.0 nm).

The biofilms inhibition percentage was determined using the following equation:^14,27,30^
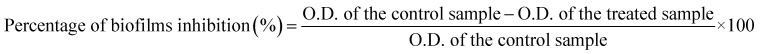


### Reaction mechanism using SEM/EDX analysis of the control and treated microbial cells

The susceptible *Candida* sp. (from the antibiofilm results; Table 4) were mixed with PBS and maintained with 3.0 ml glutaraldehyde (3.0%). Additionally, they were washed regularly with PBS and dried by different ethanol solutions (30.0%, 50.0%, 70.0%, 80.0%, 95.0%, and 100.0%) for 15 min. at room temperature (24.0 ± 2.0 °C).^30^

The prepared *Candida* cells were set up on aluminum stumps for the imaging process.^28,30^ The surface morphology of the non-treated and treated *Candida* cells with the synthesized Bi_2_O_3_ NPs-Nystatin was investigated by SEM to estimate the mode of action. The total elemental analysis of the tested *Candida* cells was estimated by EDX spectrum analysis.

### Statistical analysis

The mathematical analysis of the data was performed by ONE WAY ANOVA (at *p* < 0.05), least significant differences (LSD), and Duncan's multiple systems.^31^ The effects and data were examined and analyzed through SPSS software (version 15).

## Results and discussion

### Synthesis and optimization of Bi_2_O_3_ NPs by nystatin and gamma-rays

**Fig. 1:**
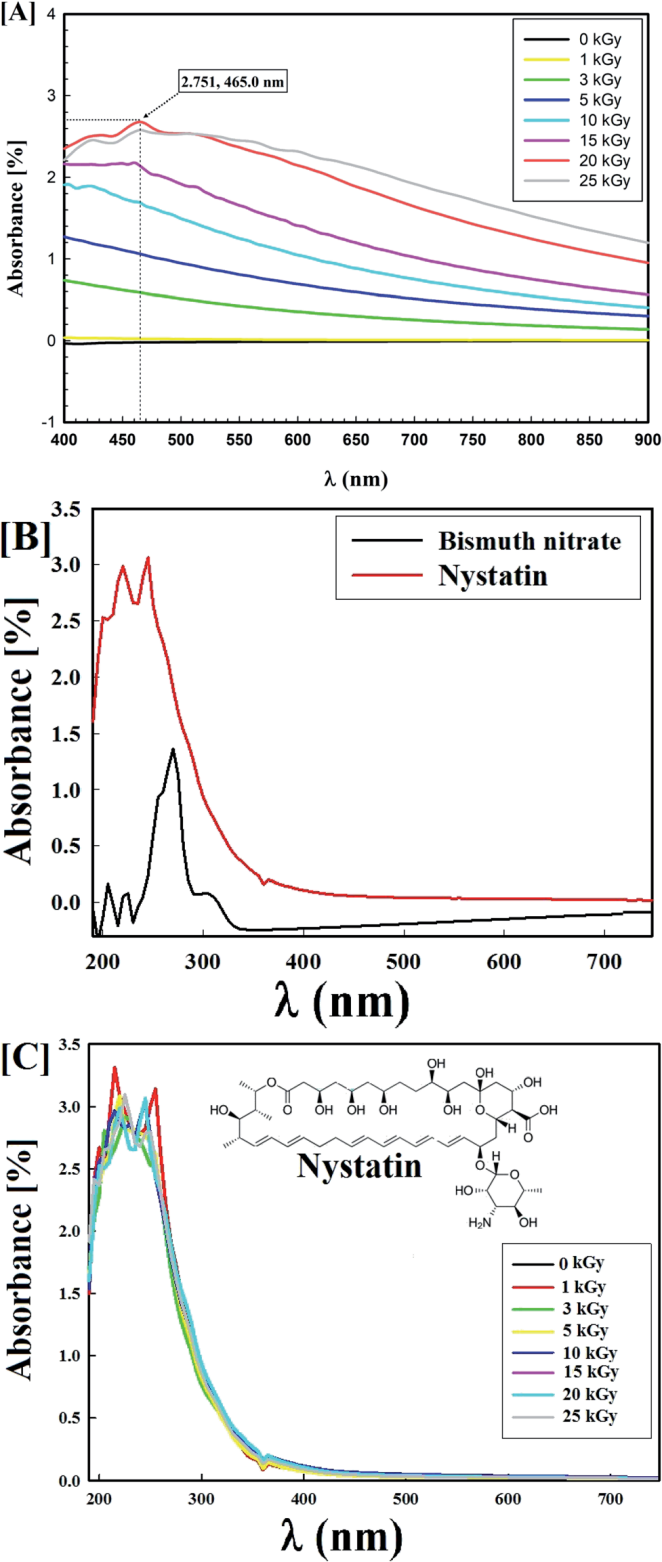
UV-Vis spectra of the synthesized Bi_2_O_3_ NPs-Nystatin, where [A] the initial screening results to obtain the most potent gamma-ray dose to be used for Bi_2_O_3_ NPs-Nystatin synthesis, [B] UV-Vis spectra of the starting materials included in the Bi_2_O_3_ NPs-Nystatin synthesis, and [C] stability of the nystatin drug after exposure to different gamma-ray doses.

The synthesized Bi_2_O_3_ NPs-Nystatin solutions were a deep off-white color due to the surface plasmon resonance (SPR) phenomenon.^32^Fig. 1A displays the optimum gamma-ray doses used for the Bi_2_O_3_ NPs synthesis, which was confirmed using a UV-Vis spectrophotometer and found to be 20.0 kGy with a high O.D. (2.751) at a wavelength of 465.0 nm.

The synthesis of Bi_2_O_3_ NPs was assisted by the reduction of various conc. of bismuth nitrate solution (0.5, 12.5, 25.0, 37.5, and 50.0 mg/10 ml) in the presence of varying nystatin conc. (0.5, 2.5, 5.0, 7.5, 10.0, and 15.0 mg/10 ml) after mixing with PVP solution (0.2%) and exposure to 20.0 kGy gamma rays.

The results listed in Table 1 show that run (4) had the optimum concentrations (2.5 mg ml^−1^ bismuth nitrate and 0.5 mg ml^−1^ NS; 1 : 5 w/w), which was reflected by a high Bi_2_O_3_ NPs yield (2.253) at 570.0 nm.

Table 1 shows that Bi_2_O_3_ NPs were not formed in runs 2 and 3, where the concentration of nystatin was set at 0.5 mg ml^−1^ and the concentration of bismuth nitrate was 0.5 and 1.25 mg ml^−1^, respectively which indicates that the concentration of bismuth nitrate was not sufficient for the Bi_2_O_3_ NPs production. In addition, there was a constant O.D. decrease from run 4 to run 6 when the nystatin concentration was set at 0.5 mg ml^−1^ and bismuth nitrate at 5.0 mg ml^−1^.

Also, Table 1 shows that there was a constant decline in the O.D. (runs 7 to 12) when the bismuth nitrate concentration was adjusted to 2.5 mg ml^−1^ and the nystatin concentration was changed from 0.25 to 1.5 mg ml^−1^, while both runs 11 and 12 involved a high concentration of nystatin, which antagonized the formation of Bi_2_O_3_ NPs.

The results confirmed that Bi_2_O_3_ NPs synthesis depended on the concentrations of bismuth nitrate and nystatin, although raising the concentration of both was ineffective. The optimum concentrations were 0.25 mg ml^−1^ of nystatin and 2.5 mg ml^−1^ of bismuth nitrate (run 8) and 0.50 mg ml^−1^ of nystatin and 2.5 mg ml^−1^ of bismuth nitrate (run 4).

On the other hand, the UV-Vis spectra of the starting materials (bismuth nitrate and nystatin) used in the Bi_2_O_3_ NPs synthesis are presented in Fig. 1B. The detectable wavelengths of both precursors were less than 300 nm, which assisted the formation of Bi_2_O_3_ NPs at the wavelength of 465.0 nm (Fig. 1A).

Fig. 1C shows the stability of the nystatin solution (0.5 mg ml; run 4) following exposure to the corresponding gamma-ray doses necessary for Bi_2_O_3_ NPs production. The results in Fig. 1C reveal that the nystatin was completely constant at all gamma-ray doses applied, which confirmed the opinion concerning the role of nystatin in the Bi_2_O_3_ NPs structure and stability. The common two peaks of nystatin appeared at 215.0 and 255.0 nm,^33^ and there was a slight decrease in the O.D. with the increase in gamma-ray doses.

This is in agreement with El-Sayyad *et al.*,^34^ who worked on the incorporation of selenium NPs with the gentamycin drug (CN) and stated that the production of Se NPs-CN nano-drug depended on the sodium selenite and CN concentrations.

### Proposal reaction mechanism for Bi_2_O_3_ NPs synthesis

When the nystatin solution (0.5 mg ml^−1^) was mixed with (2.5 mg ml^−1^) bismuth nitrate solution (the common optimized state that resulted in a great Bi_2_O_3_ NPs yield; run 4 in Table 1) and exposed to 20.0 kGy gamma rays, it produced the free-radical species (OH˙, H˙, H_2_O_2_, and H_3_O^+^) and solvated electrons (e_aq_^−^) (eqn (1)).1
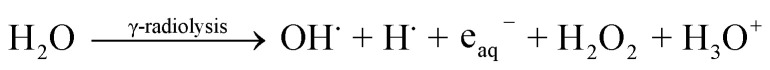


OH˙ radicals are powerful reducing agents and increase the oxidation of bismuth ions (Bi^3+^) to BiO_3_^−^ (eqn (2)).

2 2Bi^3+^ + 3OH˙ → Bi_2_O_3_^−^ + 3H^+^

The produced bismuth oxide is first dimerized while incorporated with the remaining Bi_2_O_3_^−^ and/or Bi^3+^ (eqn (3) & (4)).

3 Bi_2_O_3_^−^ + Bi_2_O_3_^−^ → 2Bi_2_O_3_^−^

4 Bi_2_O_3_^−^ + Bi^3+^ → 2Bi_2_O_3_^−^

The available radicals (OH˙ and H˙) are adequate to separate hydrogen atoms from nystatin to create nystatin free radicals (secondary radicals = 
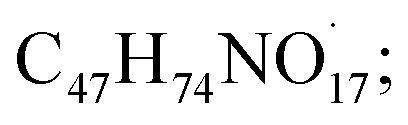
eqn (5)) then, the nystatin radicals react with Bi^3+^ to create Bi_2_O_3_ NPs and constant nystatin molecules (eqn (6)).

Finally, the formed nystatin can combine with the produced Bi^0^ (eqn (6)) though the oxygen atoms in the OH functional groups of the nystatin molecules to form more permanent Bi_2_O_3_ NPs, as displayed in eqn (7).5
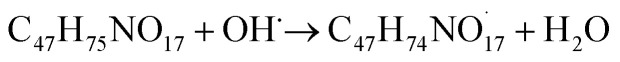
6
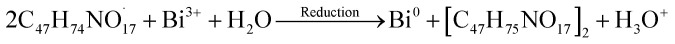
7
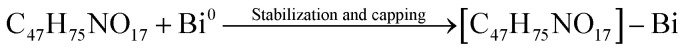


The overall reaction is related to the generation of free electrons and radical groups and the presence of notable stabilizing agents (nystatin) that drive the reduction of Bi^3+^ to Bi^0^.

### Characterization of the synthesized Bi_2_O_3_ NPs-Nystatin

#### Shape, size, and distribution of Bi_2_O_3_ NPs-Nystatin: HRTEM and DLS investigations

To investigate the common particle size and the exact shape of the incorporated Bi_2_O_3_ NPs-Nystatin, an HRTEM study was carried out and the images were correlated with the DLS results to define the average distribution of the Bi_2_O_3_ NPs-Nystatin particle size.^35^

**Fig. 2:**
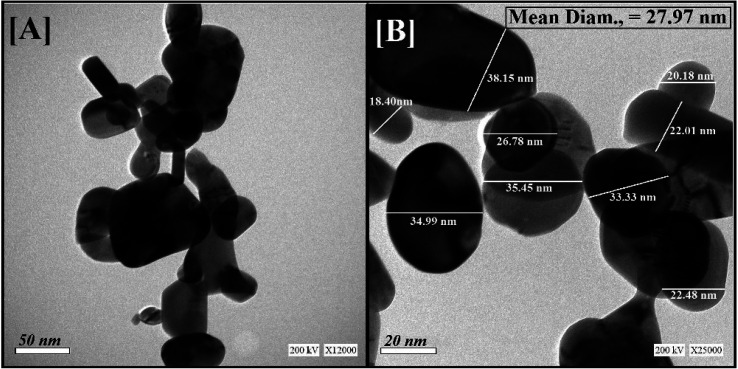
Shape, nano-structure, and mean particle-size determination by HR-TEM for Bi_2_O_3_ NPs-Nystatin, where [A] presents the low magnification (50 nm) and [B] the high magnification (20 nm) images.

The HRTEM images showed round shapes with mono-dispersed Bi_2_O_3_ NPs-Nystatin (Fig. 2A) with a range from 18.40 to 34.99 nm and the average diameter was 27.97 nm as shown in the magnified image in Fig. 2B.

**Fig. 3:**
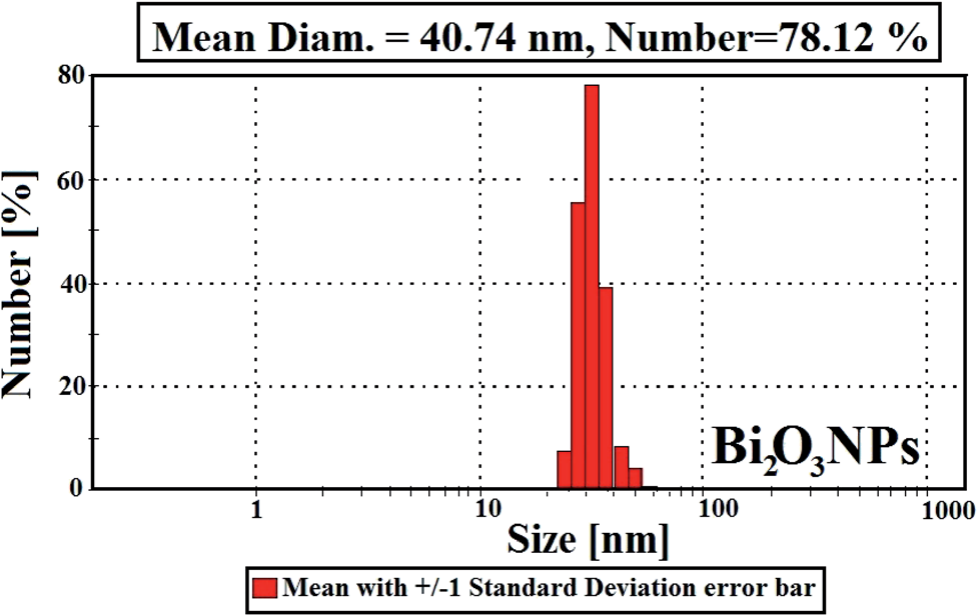
Particle-size distribution for Bi_2_O_3_ NPs-Nystatin using the DLS technique.

The average particle-size distribution was verified by the DLS method and determined as 40.74 nm in the Bi_2_O_3_ NPs incorporated with nystatin *via* applying 20.0 kGy gamma rays as shown in Fig. 3.

It was noted that the DLS size area of the incorporated Bi_2_O_3_ NPs-Nystatin was higher than the HRTEM size; this is because DLS considers the hydrodynamic diameter where the incorporated Bi_2_O_3_ NPs-Nystatin are enveloped by water molecules, leading to the greater sizes of the incorporated Bi_2_O_3_ NPs-Nystatin.^36^

#### Crystal size and crystallinity determination of the Bi_2_O_3_ NPs-Nystatin: X-ray diffraction analysis

**Fig. 4:**
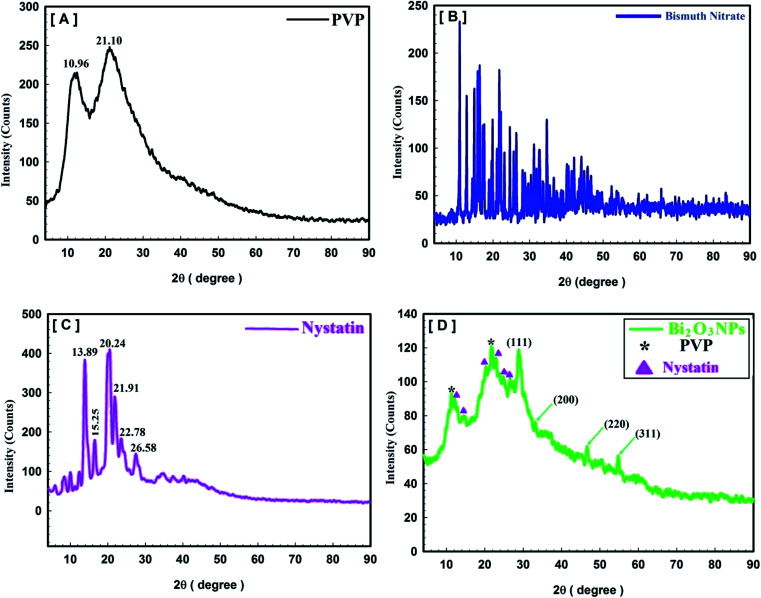
Crystallinity and crystal-size determination using XRD analysis, where [A] polyvinylpyrrolidone (PVP), [B] bismuth nitrate, [C] nystatin drug (NS), and [D] the synthesized Bi_2_O_3_ NPs-Nystatin nano-drug.

XRD was performed to determine the crystal structure and the common crystal size of the incorporated Bi_2_O_3_ NPs-Nystatin because it can show the status of the detected particles.^37^ The XRD results of the Bi_2_O_3_ NPs incorporated with nystatin after exposure to 20.0 kGy gamma rays are displayed in Fig. 4.

Fig. 4 describes the crystal and/or amorphous compositions of the starting primary materials (bismuth nitrate, PVP, and nystatin) and the synthesized Bi_2_O_3_ NPs. It must be noted that the XRD results for bismuth nitrate represent the crystal construction,^38^ as shown in Fig. 4B, and the 2*θ* at 10.96° and 21.10° were similar to the amorphous type of PVP (Fig. 4A).^39^ Additionally the 2*θ* was detected at 13.89°, 15.25°, 20.24°, 21.91°, 22.78°, and 26.58° (Fig. 4C).

The XRD data of the incorporated Bi_2_O_3_ NPs-Nystatin in Fig. 4D show the structure of the diffraction properties with 2*θ* at 28.15°, 33.65°, 46.17°, and 55.38°, which represent the Bragg's reflections at (111), (200), (220), and (311), respectively.

The presented peaks were similar with the Joint Committee on Powder Diffraction Standards (JCPDS) of Bi_2_O_3_ NPs (JCPDS file no 00-071-0465).^40^ This means that the incorporated Bi_2_O_3_ NPs-Nystatin stayed as a crystal and displayed the face-centered cubic (fcc) crystalline composition. There were additional amorphous peaks for PVP and nystatin (Fig. 4D) that was included in the assembly and stability of the Bi_2_O_3_ NPs, but their strength was less than that identified in Fig. 4A and C.

Additionally, the average crystallite size of the incorporated Bi_2_O_3_ NPs-Nystatin was defined by applying the Williamson–Hall (W–H) equation,^41,42^ and was observed to be 30.54 nm for Bi_2_O_3_ NPs produced by nystatin *via* 20.0 kGy gamma-ray application according to eqn (8).8
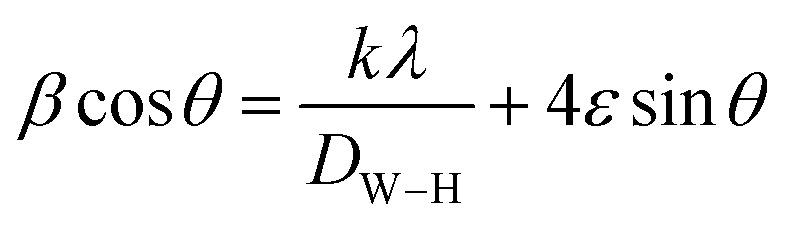
where *D*_W–H_ is the average crystallite size, *λ* is the X-ray wavelength, *β* is the full-width at half maximum, *ε* is the strain of the samples, *θ* is the Bragg's angle, and *k* is a constant.

#### Surface morphology and elemental composition of Bi_2_O_3_ NPs-Nystatin: SEM and EDX analyses

**Fig. 5:**
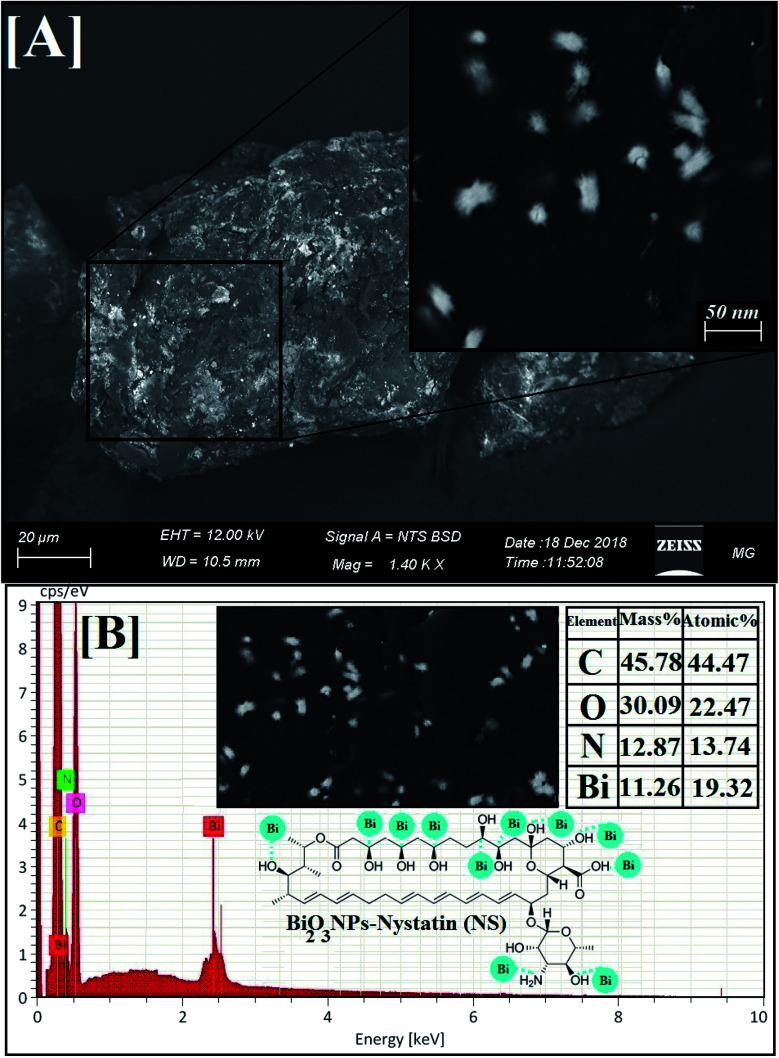
Surface morphology and elemental analysis of the synthesized Bi_2_O_3_ NPs-Nystatin, where [A] SEM image, [B] EDX elemental analysis.

The surface characteristics and morphology of the incorporated Bi_2_O_3_ NPs-Nystatin were investigated by SEM technique. Fig. 5A illustrates the SEM image of the synthesized Bi_2_O_3_ NPs-Nystatin at 20.0 kGy gamma-ray irradiation including the different grain sizes and the equivalent round shape. It can be noticed that the Bi_2_O_3_ NPs were distributed beyond the nystatin drug, which shows them as brilliant NPs linked near by the antibiotic units.

EDX is an analytical method used for the elemental investigation or the qualitative chemical validation of synthesized metal oxide NPs.^37,42^ EDX analysis was used to establish the elemental composition of the synthesized Bi_2_O_3_ NPs-Nystatin and its capacity for determining the purity of the synthesized Bi_2_O_3_ NPs as represented in Fig. 5B.

The synthesized Bi_2_O_3_ NPs exhibited notable absorption peaks related to the bismuth element at 0.25 and 2.35 keV. The lack of further elemental peaks and a large amount of bismuth in the spectra confirmed the purity of the bismuth element. The presence of carbon, oxygen, and nitrogen peaks in the examined samples was due to the presence of stabilizers or the capping factor (nystatin drug, Fig. 5B).

#### Mapping analysis of the elements in Bi_2_O_3_ NPs-Nystatin

**Fig. 6:**
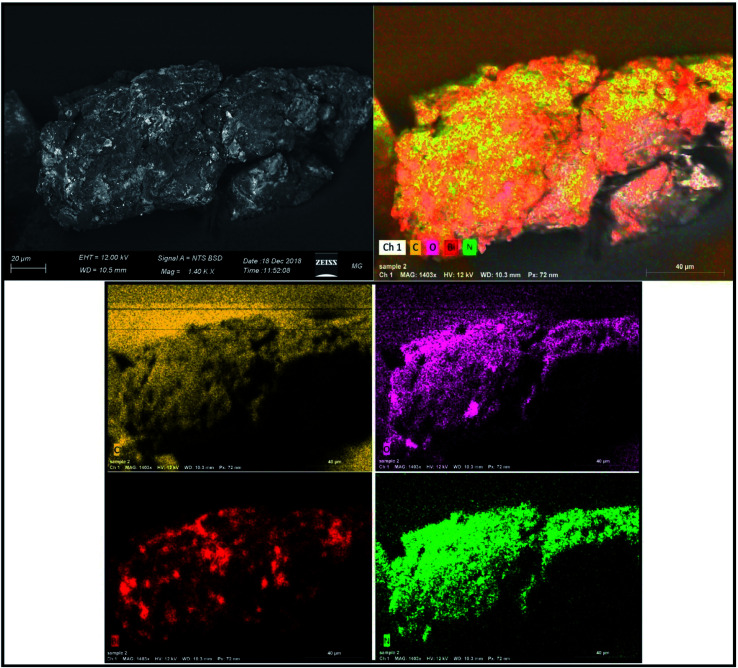
SEM/EDX mapping images of the synthesized Bi_2_O_3_ NPs-Nystatin.

The mapping models of the elements present in Bi_2_O_3_ NPs-Nystatin are displayed in Fig. 6. The images are defined as C, O, Bi, and N for the incorporated Bi_2_O_3_ NPs-Nystatin.

The displayed atoms were related to the distribution to Bi, C, O, and N atoms. Moreover, the C, O, and N atoms were in agreement with the nystatin drug. Subsequently according to the given image, the synthesized Bi_2_O_3_ NPs-Nystatin (bright red NPs) was developed regularly across the nystatin atoms (C, O, and N).

#### Surface bonding and functional groups analysis; FTIR analysis of Bi_2_O_3_ NPs-Nystatin

**Fig. 7:**
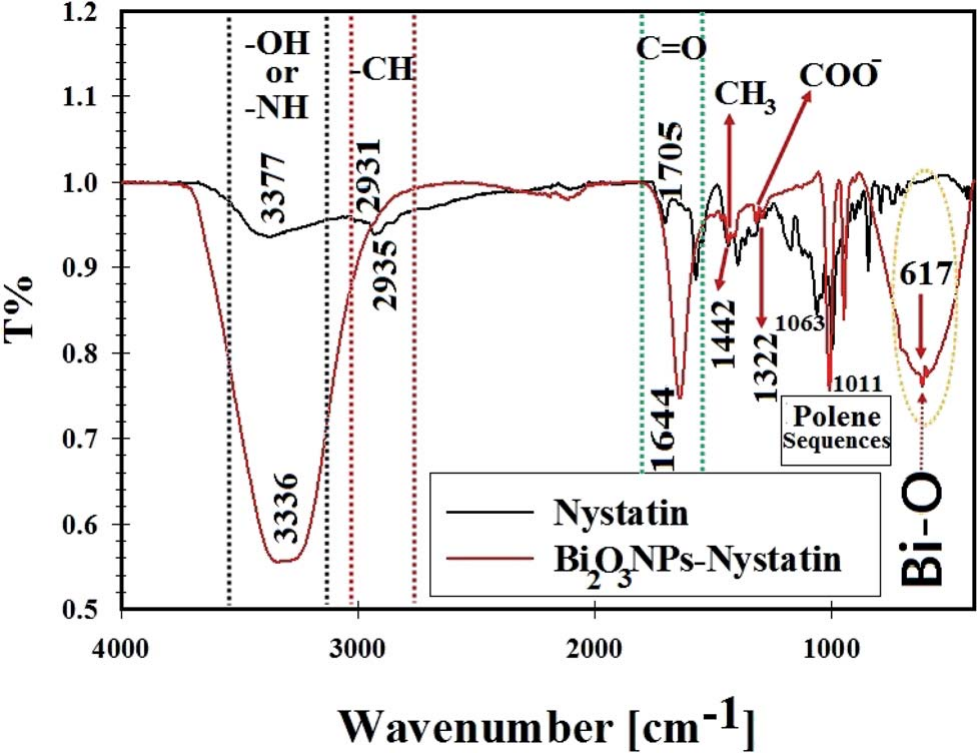
FT-IR spectra, surface bonding, and functional groups analysis for nystatin (NS) and the synthesized Bi_2_O_3_ NPs-Nystatin.

FTIR investigations were performed to determine the interaction between Bi_2_O_3_ NPs and the antifungal nystatin drug (Fig. 7). The FTIR spectrum of the nystatin drug had absorption bands at 3377.20, 2935.12, 1705.36, 1442.56, 1322.0, and 1063.84 cm^−1^, and the absorption bands for the synthesized Bi_2_O_3_ NPs-Nystatin were at 3336.16, 2931.52, 1644.88, 1442.55, 1322.0, 1011.08, and 617.44 cm^−1^, as shown in Fig. 7.

The broad peak at 3336.16 cm^−1^ was assigned to the –OH of the hydroxyl group and –NH stretching, while the peak at 2931.52 cm^−1^ was attributed to the asymmetric and symmetric –CH vibrations of the –CH_2_ group. The peak at 1644.88 cm^−1^ was related to –C=O stretching of the ester group.

The peak located at 1442.55 cm^−1^ was designated to –CH_3_. A further band at 1322.0 cm^−1^ was related to –COO^−^. Also, the peaks located at 1011.08 cm^−1^ were due to polyene sequences. A definite peak at 617.44 cm^−1^ was identified in the FTIR of Bi_2_O_3_ NPs-Nystatin, which may be associated with the conjugation and attraction of Bi_2_O_3_ NPs beside the hydroxyl group in the nystatin drug as Bi–O.^37^ The FTIR results in the present study were similar to those in recently published research studies.^23,43,44^

According to the FTIR results in the present research, it was concluded that the intensity of all the detected peaks was reduced in the FTIR of Bi_2_O_3_ NPs-Nystatin. This may be because of the interaction of Bi_2_O_3_ NPs, the –OH, and other functional groups present in the nystatin drug.

It was noted that the nystatin drug may combine with Bi_2_O_3_ NPs either by the available amine residue and/or by the electrostatic attraction among the carboxylate groups, which hold a negative charge^45^ so they support the Bi_2_O_3_ NPs from aggregation through the influence of the O and/or N atoms present in the nystatin drug.

#### *In vitro* antimicrobial activity of the synthesized Bi_2_O_3_ NPs-Nystatin

**Fig. 8:**
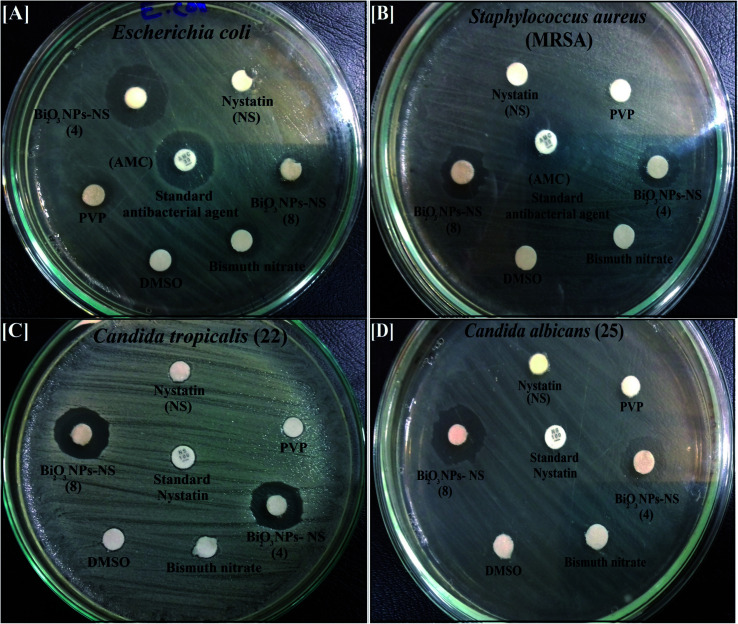
Antimicrobial activity of the synthesized Bi_2_O_3_ NPs-Nystatin (two different samples; see Table 2), nystatin, bismuth nitrate, DMSO, and PVP against [A] *Escherichia coli*, [B] *Staphylococcus aureus*, [C] *Candida tropicalis* (22), and [D] *Candida albicans* (25) as ZOI.

It was obvious from the disc agar distribution method (as a screening procedure) that the incorporated Bi_2_O_3_ NPs-Nystatin displayed a qualitative antimicrobial potential toward all the tested bacterial strains and *Candida* pathogens. The *in vitro* ZOI result verified that the Bi_2_O_3_ NPs-Nystatin exhibited an elevated antibacterial activity against *E. coli* (17.0 mm ZOI; Fig. 8A) and *S. aureus*; MRSA (13.0 mm ZOI; Fig. 8B), as displayed in Table 2.

**Table 2:** Antibacterial and antifungal activities of mono-dispersed Bi_2_O_3_ NPs-Nystatin, PVP, DMSO, and its precursors (bismuth nitrate and nystatin) against some multi-drug-resistant (MDR) bacteria and pathogenic *Candida* species as ZOI (mm) and MIC (μg ml^−1^)

Pathogenic microbes	ZOI of Bi_2_O_3_ NPs-NS (run 4) (1 mg NS : 5 mg Bi) (mm)	MIC of Bi_2_O_3_ NPs-NS (run 4) (NS : Bi; μg ml^−1^)	ZOI of Bi_2_O_3_ NPs-NS (run 8) (0.5 mg NS : 5 mg Bi) (mm)	ZOI of bismuth nitrate (mm)	ZOI of nystatin (mm)	ZOI of PVP (mm)	ZOI of DMSO (mm)	⁃AMC or NS
*Candida albicans* (1)	14.0^ab^ ± 0.5773	1.95 : 4.2	13.0^de^ ± 0.5773	Nil	Nil	Nil	Nil	Nil
*Candida albicans* (25)	15.0^c^ ± 0.5773	0.24 : 0.52	13.0^bc^ ± 0.5773	Nil	Nil	Nil	Nil	7.0^a^ ± 0.5000
*Candida albicans* (33)	14.0^bc^ ± 0.5773	0.48 : 1.05	12.0^bc^ ± 0.2886	Nil	Nil	Nil	Nil	Nil
*Candida tropicalis* (1)	15.0^bc^ ± 0.5773	0.24 : 0.52	13.0 ^cd^ ± 0.7637	Nil	Nil	Nil	Nil	7.0^a^ ± 0.2886
*Candida tropicalis* (22)	15.0^c^ ± 0.5773	0.48 : 1.05	14.0^e^ ± 1.1547	Nil	7.0	7.0	Nil	7.0^a^ ± 0.7637
*Escherichia coli*	17.0^d^ ± 0.5773	1.95 : 4.2	12.0^bc^ ± 0.5000	8.0	Nil	Nil	7.0	17.0^c^ ± 0.2886
*Pseudomonas aeruginosa*	•Nil	3.9 : 8.4	Nil	Nil	Nil	Nil	Nil	Nil
*Staphylococcus aureus*; MRSA	13.0^a^ ± 0.5773	0.24 : 0.52	12.0^b^ ± 0.6110	Nil	Nil	Nil	Nil	15.0^b^ ± 0.5000
*Bacillus cereus*	13.0^a^ ± 0.5773	0.24 : 0.52	10.0^a^ ± 0.4509	Nil	Nil	Nil	Nil	7.0^a^ ± 0.4618
LSD	1.00000	—	1.33333	—	—	—	—	1.83333

a Values arethe mean ± SD (*n* = 3). Data within the groups were analyzed using one-way analysis of variance (ANOVA) followed by ^a–e^ Duncan's multiple range test (DMRT), LSD = least significant differences. •Nil means that no ZOI had been measured. ⁃AMC = amoxicillin/clavulanic acid (antibacterial standard), NS = nystatin (antifungal standard).

It worth noting that the antibacterial potency of the Bi_2_O_3_ NPs-Nystatin was significantly more powerful than bismuth nitrate, PVP, DMSO, the nystatin drug alone, and the standard antimicrobial agents (AMC).

It is also necessary to note that the synthesized Bi_2_O_3_ NPs-Nystatin were active against Gram-negative bacteria more than Gram-positive. Note, the cell wall constituents in Gram-negative bacteria contain principally little layers of lipopolysaccharide, lipid, and peptidoglycan. On the other hand, the cell wall of Gram-positive incorporate very solid peptidoglycan forms.^46^

Additionally, the synthesized Bi_2_O_3_ NPs-Nystatin were shown to incorporate promising antifungal factors as they exhibited tremendous antifungal efficiency against *C. tropicalis* (22) (15.0 mm ZOI; Fig. 8C) and *C. albicans* (25) (15.0 mm ZOI; Fig. 8D), as recorded in Table 2.

There is a relationship between the characteristics of the synthesized Bi_2_O_3_ NPs-Nystatin and the antimicrobial effects discussed. The Bi_2_O_3_ NPs-Nystatin were stable because of the PVP polymer applied, their reduced crystal size (30.54 nm; Fig. 4D), and separated spherical form with a particle size within the nano-scale (27.97 nm; Fig. 2), as well as their uniformity (EDX; Fig. 5B) and pattern of mono-dispersed highly-distributed NPs (40.74 nm; Fig. 3), which served as an essential objective for enhancing the antimicrobial potency of the Bi_2_O_3_ NPs-Nystatin at low concentration (1 : 5 w/w), against all the tested bacterial and *Candida* sp.

The Bi_2_O_3_ NPs-Nystatin displayed individual physical and chemical properties better than the traditional organic and synthesized antimicrobial agents, such as decreased crystal sizes, reduced average particle size, more stability, and a higher potency for interaction with more pathogenic bacteria and *Candida* sp., thus consequently, increasing their antimicrobial potential.^37^

The MIC results of the Bi_2_O_3_ NPs-Nystatin against all the tested pathogenic bacteria and *Candida* sp. ranged from 3.9 μg ml^−1^ nystatin: 8.4 μg ml^−1^ Bi_2_O_3_ NPs, to 0.24 μg ml^−1^ nystatin: 0.52 μg ml^−1^ Bi_2_O_3_ NPs, as mentioned in Table 2. The Bi_2_O_3_ NPs-Nystatin possessed a promising MIC of 0.24 μg ml^−1^ nystatin: 0.52 μg ml^−1^ Bi_2_O_3_ NPs against *S. aureus*; MRSA, B. cerus, *C. albicans* (25), and *C. tropicalis* (1).

The Bi_2_O_3_ NPs-Nystatin's size was not the only parameter indicating the antimicrobial characteristics, but other features, such as their mono-dispersity, simplicity, stability, and their appearance, should be considered.

The results from similar studies^23,47–49^ of the antimicrobial behavior of the incorporated Bi_2_O_3_ NPs-Nystatin against some bacteria and fungi-causing infectious diseases are introduced in Table 3. The encouraging antimicrobial potential of the synthesized Bi_2_O_3_ NPs-Nystatin in our research was due to their small particle and/or crystal sizes, extensive purity, superior stability by using the PVP polymer, and the incorporation with the nystatin drug, which enhanced their synergistic impact.

**Table 3:** Comparative antimicrobial studies between different synthesized Bi_2_O_3_ NPs

Methods of Bi_2_O_3_ NPs preparation	Average particle size (nm)	Starting concentration (μg ml^−1^)	Antimicrobial activity (ZOI; mm) and/or (MIC; μg ml^−1^)	References
Green synthesis using nystatin (NS) drug and gamma rays	27.97 nm	1 mg NS : 5 mg Bi/ml for ZOI and MIC	15.0 mm ZOI and 0.24 NS: 0.52 Bi μg ml^−1^ MIC	Our research
Biogenic synthesis by fungal melanin pigment and gamma rays	29.82 nm	0.8 μg ml^−1^ for ZOI and MIC	20.0 mm ZOI and 0.4 μg ml^−1^ MIC	23
Chemical synthesis of bismuth dimercaptopropanol nanoparticles (BisBAL NPs)	18.7 nm	0.1–100 μM for ZOI and MIC	BisBAL NPs inhibited *S. mutans* and *S. gordonii* growth by more than 70% at 0.1 μM and, MIC of for *S. mutans*, *S. gordonii* was 5 μM, and *C. albicans* was 10 μM	47
Biological synthesis of bismuth nanoparticles by *Delftia* sp.	40–120 nm	1000 μg per disc for ZOI	27.0 mm ZOI and 1280 μg ml^−1^ MIC	48
Biosynthesis of elemental bismuth oxide nanoparticles by *Serratia marcescens*	≤5 nm	40–140 μg ml^−1^	100 μg ml^−1^ MIC	49

#### The antibiofilm activity of the incorporated Bi_2_O_3_ NPs-Nystatin

Biofilm production was identified in various microbes in the absence and presence of Bi_2_O_3_ NPs-Nystatin as assessed by the tube technique.^28,30^

**Fig. 9:**
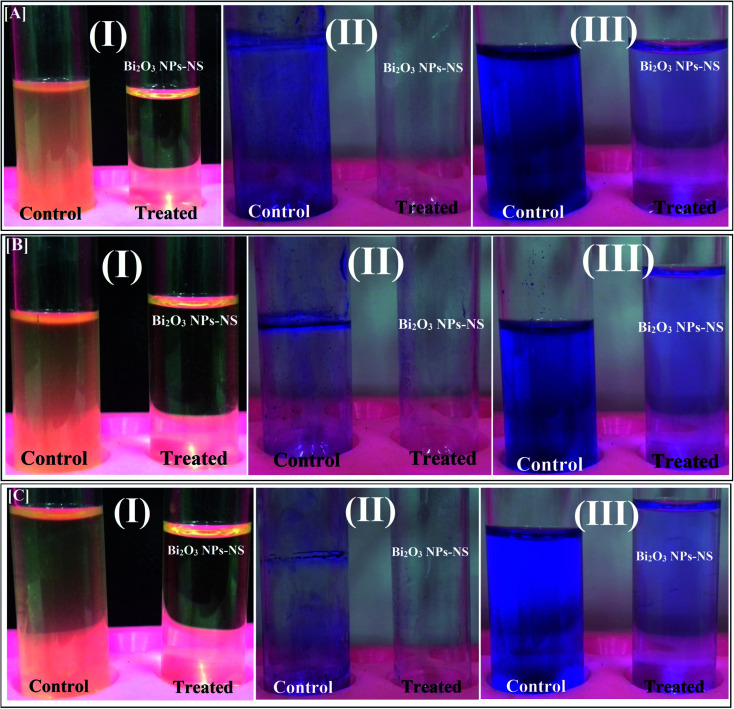
Antibiofilm activity of the synthesized Bi_2_O_3_ NPs-Nystatin using the tube method against [A] *Candida albicans* (1) [B] *Escherichia coli*, and [C] *Bacillus cereus*, where the steps are reported as follows: (I) growth of the bacterial and yeast cells and biofilm formation (rings) without treatment with the synthesized Bi_2_O_3_ NPs-Nystatin (NS) and the inhibition of bacterial and yeast growth after treatment with Bi_2_O_3_ NPs-Nystatin; (II) staining of the adherent bacterial and yeast cells with crystal violet and (III) removing and dissolving the adherent bacterial and yeast cells by ethanol for semi-quantitative biofilm inhibition (%) determination (as shown in Table 4).

*C. albicans* (1) in the absence of Bi_2_O_3_ NPs-Nystatin formed a thick whitish-yellow matt across the air–liquid interface, which adhered entirely to the tube walls and gave a blue color when stained with crystal violet. Also, a dark blue suspension was formed following dissolving the CV by pure ethanol, as presented in Fig. 9A.

The *C. albicans* (1) tubes treated with Bi_2_O_3_ NPs-Nystatin (1.95 NS: 4.2 Bi; μg ml^−1^), revealed a negative biofilm formation. The color of the adherent cells was light blue after CV staining, as shown in Fig. 9A. The same conditions were described for the biofilm repression of *E. coli* and *Bacillus cereus*, as displayed in Fig. 9B and C, respectively.

To determine the inhibition percentage (%) of the biofilm created by the examined pathogens, a UV-Vis spectrophotometer (set at 570.0 nm) was applied. The O.D. estimated the subsequent dissolving of the stained biofilm through ethanol.^29^

Table 4 records the repression percentages of the biofilm generation by the tested bacteria and *Candida* sp. The highest restraint% was noted for *C. albicans* (1) (94.15%), followed by *E. coli* (84.85%), and *B. cereus* (84.79%) after treatment with Bi_2_O_3_ NPs-Nystatin (1.95 NS: 4.2 Bi; μg ml^−1^).

**Table 4:** Semi-quantitative inhibition% of the biofilm formation for non-treated and treated pathogenic bacteria and *Candida* species with Bi_2_O_3_ NPs-Nystatin^a^

Pathogenic microbes	O.D. of crystal violet staining at 570.0 nm (control)	O.D. of crystal violet staining at 570.0 nm (treated with Bi_2_O_3_ NPs-Nystatin; run 4)	Inhibition%
*Candida albicans* (1)	2.548^h^ ± 0.0017	0.150^c^ ± 0.0005	94.15%
*Candida albicans* (25)	0.850^e^ ± 0.0010	0.560^g^ ± 0.0113	34.17%
*Candida albicans* (33)	0.669^c^ ± 0.0023	0.127^a^ ± 0.0011	81.01%
*Candida tropicalis* (1)	0.495^a^ ± 0.0023	0.165^d^ ± 0.0015	66.66%
*Candida tropicalis* (22)	0.514^b^ ± 0.0010	0.201^f^ ± 0.0015	60.89%
*Escherichia coli*	0.977^f^ ± 0.0005	0.148^c^ ± 0.0010	84.85%
*Pseudomonas aeruginosa*	2.767^i^ ± 0.0060	0.905^h^ ± 0.0026	67.25%
*Staphylococcus aureus*; MRSA	0.748^d^ ± 0.0023	0.137^b^ ± 0.0020	81.68%
*Bacillus cereus*	1.217^g^ ± 0.0015	0.185^e^ ± 0.0010	84.79%
LSD	0.01273	0.01267	—

a Values are the mean ± SD (*n* = 3). Data within the groups were analyzed using one-way analysis of variance (ANOVA) followed by ^a–i^ Duncan's multiple range test (DMRT), and LSD = least significant differences.

The synthesized Bi_2_O_3_ NPs-Nystatin was applied to repress biofilm development in its constant adhesion step (also identified as the primary stage).^50^ However, the mechanistic behavior of the incorporated Bi_2_O_3_ NPs upon biofilm development has yet to be confirmed.

The difference in the inhibitory percentage may be described by many aspects, like antimicrobial potency, biosorption (due to the high surface area of the incorporated Bi_2_O_3_ NPs-Nystatin), physical features (Bi_2_O_3_ NPs-Nystatin size; 27.97 nm), penetration capabilities, and distinct chemical attributes that influence the relationship and synergy of the Bi_2_O_3_ NPs-Nystatin with the *Candida* biofilm.^51^

It was clear that the Bi_2_O_3_ NPs-Nystatin restrained *C. albicans* biofilm expansion by a factor of more than 98% at (1.95 NS: 4.2 Bi; μg ml^−1^) Bi_2_O_3_ NPs-Nystatin (MIC results; Table 2). The exopolysaccharide (the principal precursors of biofilm production) formation was hindered so *C. albicans* could not produce a biofilm.^28^ Our anti-biofilm research is comparable to the findings of Ashajyothi *et al.*,^50^ who stated that the synthesized ZnO NPs displayed a biofilm hindrance% of 10.7% against *P. aeruginosa* following 18 h incubation.

#### Bi_2_O_3_ NPs-Nystatin mode of action against *Candida* sp. using SEM/EDX technique

Moreover, to describe the antibiofilm impact of Bi_2_O_3_ NPs-Nystatin, we suggested a reaction mechanism for the synthesized Bi_2_O_3_ NPs-Nystatin toward *C. albicans* (1) that produced a biofilm. The mode of action was investigated using SEM and EDX examination.^52^ Through the SEM technique, the *Candida* cell morphologies could be observed in the cases of the non-treated and treated cells with the Bi_2_O_3_ NPs-Nystatin.

**Fig. 10:**
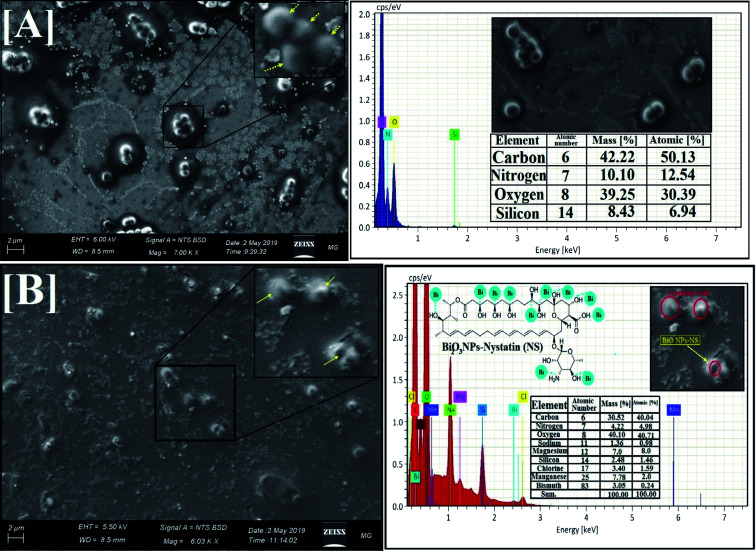
SEM images and the corresponding EDX elemental analysis of *Candida albicans* (1), where [A] normal *Candida* cells without Bi_2_O_3_ NPs-Nystatin treatment (yellow arrows for normal budded *Candida* cells), and [B] depressed, and deformed *Candida* cells under the influence of Bi_2_O_3_ NPs-Nystatin (yellow arrows represent the complete lysis of the *Candida* cells and loss of budding formation).

Initially, the yeast populations (control without Bi_2_O_3_ NPs-Nystatin treatment) were developed regularly and showed the definite normal cellular morphology with the usual cell surface, budding appearance, and a developed biofilm, as explained in Fig. 10A.

By comparison, the morphological modifications were recognized in *C. albicans* (1) cells following treatment with the incorporated Bi_2_O_3_ NPs-Nystatin at 1.95 NS: 4.2 Bi μg ml^−1^ (Fig. 10B). An obvious surface cell break and consequent deformation and failure to form budding properties of the treated *C. albicans* (1) upon Bi_2_O_3_ NPs-Nystatin addition were noted. Furthermore, the number of viable cells and biofilm production were repressed. SEM analysis revealed that the Bi_2_O_3_ NPs-Nystatin were directed to effect *Candida* cell wall depreciation (Fig. 10B).^52^ The EDX elemental study showed the appearance of Bi and O at the shrinking cell membrane and on the outside surface of the treated *C. albicans* (1), which confirmed Bi_2_O_3_ NPs-Nystatin action toward the yeast cells (Fig. 10B; inset).

One potential reason for Bi_2_O_3_ NPs-Nystatin action upon *C. albicans* (1) could be connected to the elevated surface area of the Bi_2_O_3_ NPs-Nystatin providing more reliable interactions among the negatively-charged *Candida* cell walls, as shown in Fig. 10B. Additional studies revealed that the metal oxide NPs combine with pathogens by their electrostatic potential and defeat bacteria by layer separation.^53^

A recent report examined the interaction between Bi_2_O_3_ NPs and the tested microbes and found that the attraction takes place by electrostatic attraction leading to the membrane leakages.^53^ Further research revealed that the metal oxide NPs attack the microbes and increase the oxidative pressure,^54^ which quickly changes the yeast cells due to the high level of ROS production. The free radicals generation is influenced by the prominent reduction in oxygen by the electron change above the oxygen atom through electron transport in the mitochondria.^53^

**Fig. 11:**
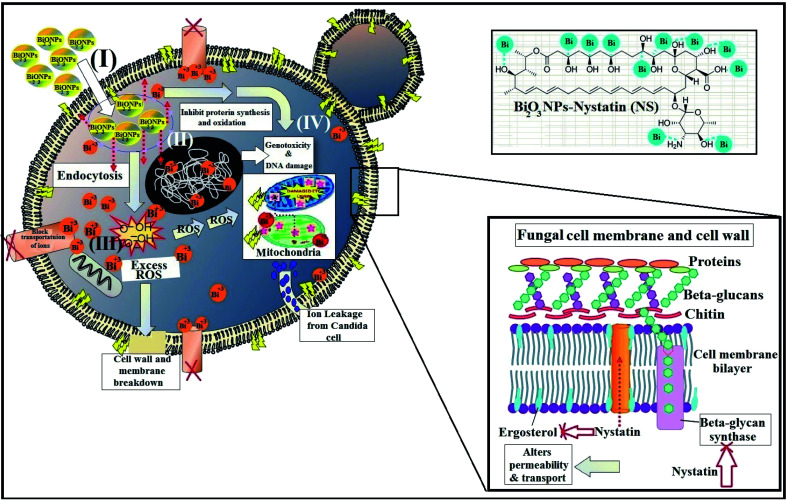
The four common mechanisms of the antimicrobial activity of Bi_2_O_3_ NPs-Nystatin, where (I) Bi_2_O_3_ NPs adhere to the surface of the pathogenic *Candida* cell and affect the membrane structure and penetrate the cell membrane due to their small size, (II) Bi_2_O_3_ NPs diffuse inside the *Candida* cells and associate with *Candida* organelles and bio-molecules, thereby changing the cellular mechanism and producing genotoxicity, (III) Bi_2_O_3_ NPs create ROS inside the *Candida* cells, which lead to the cell destruction, and (IV) Bi_2_O_3_ NPs change the cellular sign order, eventually inducing cell necrosis. Additionally, the Bi_2_O_3_ NPs may assist as a carrier to release Bi^3+^ ions more efficiently to the *Candida* cytoplasm and layer, in which the proton motive force may reduce the pH (below pH 3.5), which improves Bi^3+^ ions release. Nystatin alters the action of beta-glycan synthase and ergosterol construction and subsequently alters the permeability of the cell membrane and the transportation of ions inside the *Candida* cells.

It must be mentioned that the suggested reaction mechanism of metal oxide NPs toward the pathogenic bacteria and *Candida* cells was explained in our earlier studies^14^ and is schematically drawn in Fig. 11.

The mode of action included four mechanisms that describe the effect of Bi_2_O_3_ NPs-Nystatin toward *Candida* cells. It begins with the adhesion of Bi_2_O_3_ NPs-Nystatin near the surface of the *Candida* cells. Following that, Bi^3+^ ions inside the *Candida* cells split the intracellular organic molecules (DNA and mitochondria). Then the cellular toxicity, like oxidative stress, is created by the formation of ROS. Finally, Bi_2_O_3_ NPs-Nystatin stimulates the *Candida* signal transduction pathways, in addition to the performance of nystatin which alters the action of beta-glycan synthase and ergosterol construction and subsequently alters the permeability of the cell membrane and the transportation of ions inside the *Candida* cells.^55^

#### Toxicity of the Bi_2_O_3_ NPs

Nano-materials have attracted much attention in diverse areas extending from biomedicine to manufacturing due to their outstanding physicochemical characteristics and purposes, leading to spreading human susceptibility to different NPs. Bismuth-based composites have been generally accepted for technical, pharmaceutical and biomedical purposes. Although the toxicity of the bismuth composites is considered at times, there is a severe absence of data regarding their toxicity and outcomes in the nano-scale on personal health and the climate.^56^

The genotoxic effects of Bi_2_O_3_ NPs at various concentrations (12.5, 25.0, 50.0, 75.0, and 100.0 μg ml^−1^) were investigated on the root cells of *Allium cepa*. The results indicated that the exposure to Bi_2_O_3_ NPs considerably enhanced the mitotic index (except at 12.5 μg ml^−1^) and the total chromosomal differences, while confused anaphase–telophase, anaphase bridges, and stickiness chromosome laggards were recognized in anaphase–telophase cells. A notable improvement in DNA destruction was also recognized at all Bi_2_O_3_ NPs concentrations (except at 12.5 μg ml^−1^).^57^

In another study,^56^ the toxic impacts of Bi_2_O_3_ NPs on the liver (HepG2 cell), intestine (Caco-2 colorectal cell), kidney (NRK-52E epithelial cell), and lung (A549 lung cell) were studied. It was mentioned that Bi_2_O_3_ NPs reduced the cell viability by intruding on the mitochondrial and lysosomal purposes in HepG2, Caco-2, NRK-52E, and A549 cells in a dose-subject way. The IC_50_ values of Bi_2_O_3_ NPs were counted at 35.10–96.50 μg ml^−1^.

Furthermore, Kovriznych *et al.*^58^ stated that the acute toxic amount (LC_50_ evaluation) of Bi_2_O_3_ NPs was less than 1.6 μg ml^−1^ in adult fish and zebra fish eggs. The cytotoxicity of Bi_2_O_3_ NPs may be associated with several distinct agents, like oxidative destruction in living systems.

New studies report that Bi_2_O_3_ NPs influence oxidative stress by elevating reactive oxygen species, membrane lipid peroxidation, and reducing intracellular glutathione (GSH).^59^ Additionally, Yang Luo *et al.*^60^ reported that Bi NPs are non-toxic at a concentration of 0.5 nM, but at elevated concentration (50 nM) they induced cytotoxicity and killed about 45% of HeLa cells.

Our study assumed that the synthesized Bi_2_O_3_ NPs-Nystatin could serve as a bactericidal and strong fungicidal agent, and would not create any deadly impact on the human system. Animal kidney cells were exposed to 2 mM Bi_2_O_3_ NPs for one day and their cytotoxic influence was not verified.^22^ Although the Bi_2_O_3_ NPs represent a pleasant approach to reduce infections, more investigation is needed to ensure their proper application for human society.^23^

The interesting thing about inorganic NPs are their high surface-to-volume degrees, many structural advantages, several applications, and nano-scale size, which represent infinite dynamic forms to connect with living forms, like pathogenic bacteria and fungi. This is the significant difference between different NPs and traditional organic antimicrobial agents, which in turn could help minimize the risk of developing antimicrobial resistance.^23^

The results obtained in this study propose unique, efficient, low-cost, and broad-spectrum antimicrobial factors. The Bi_2_O_3_ NPs could be used at low concentration in foodstuff, pharmaceutical applications, dental supplements, labs, disinfectant, clinics, and geriatric and pediatric hospitals primarily for *Candida* sp. infection treatment.

## Conclusion

In this study, the synergistic effect of Bi_2_O_3_ NPs and nystatin to govern “*Candida*” germination and biofilms creation was verified. The study also reported an encouraging method to restrain the pathogenic *Candida* sp. and some examined bacteria using Bi_2_O_3_ NPs-Nystatin. This planned method could not only dramatically reduce the administered nystatin dose but could also improve its potency. An eco-friendly and cost-efficient approach was applied to synthesize the Bi_2_O_3_ NPs using nystatin drug and polyvinylpyrrolidone (PVP) as stabilizing agents after exposure to 20.0 kGy gamma-ray irradiation. Joining Bi_2_O_3_ NPs with the nystatin antifungal agent is a modular approach that could be implemented as a strategy for enhancing the currently ineffective nystatin drug. The average particle size and surface morphology of the incorporated Bi_2_O_3_ NPs-Nystatin were exhibited to be mono-dispersed and rounded with an average size of 27.79 nm. EDX elemental analysis confirmed the growth of pure Bi_2_O_3_ NPs-Nystatin without any impurities. In addition, the antimicrobial potential in terms of ZOI and MIC toward different *Candida* sp. and some pathogenic bacteria was studied. The incorporated Bi_2_O_3_ NPs-Nystatin at low concentration (MIC = 0.24 : 0.52; NS : Bi μg ml^−1^) restrained the development and attack of *C. albicans*. The current study considers that the small crystal size (≈30 nm) and high purity and stability play the main roles in the success of the combination of Bi_2_O_3_ NPs-Nystatin, at reduced concentrations, toward all the examined bacteria and *Candida* cells. Furthermore, the antibiofilm potential of the incorporated Bi_2_O_3_ NPs-Nystatin was extremely encouraging (94.15% inhibition toward *C. albicans* (1)). The morphological modifications of the *Candida* cells after treatment with Bi_2_O_3_ NPs-Nystatin (at a ratio of 1 : 5 w/w) were conceived as noticeable alterations in the cell hardness and visible surface cell brokenness. Also, the consequent deformation and loss of budding features of *C. albicans* (1) were limited after treatment with the synthesized Bi_2_O_3_ NPs-Nystatin. The novel synthetic process for the Bi_2_O_3_ NPs-Nystatin is a promising method for possible use in manufacturing, pharmaceutical, and biomedical purposes and for managing dangerous infections, particularly candidoses. However, future work in this area needs to continue to cover the safety of using these small-sized materials and their activity over the course of application. In addition, investigation of the mechanism of the interactions across the genetic level of this type of nano-drug with current and other types of bacteria and pathogenic fungal strains is an essential element needed to complete the work.

## Conflicts of interest

The authors declare that they have no conflict of interests.

## Supplementary Material
